# Timing and duration of lipofection-mediated CRISPR/Cas9 delivery into porcine zygotes affect gene-editing events

**DOI:** 10.1186/s13104-021-05800-8

**Published:** 2021-10-09

**Authors:** Qingyi Lin, Quynh Anh Le, Koki Takebayashi, Chommanart Thongkittidilok, Manita Wittayarat, Maki Hirata, Fuminori Tanihara, Takeshige Otoi

**Affiliations:** 1 Bio-Innovation Research Center, Tokushima University, Tokushima, Japan; 2 Faculty of Bioscience and Bioindustry, Tokushima University, Tokushima, Japan; 3 Faculty of Veterinary Science, Prince of Songkla University, Songkhla, Thailand; 4 Present Address: Center for Development of Advanced Medical Technology, Jichi Medical University, Tochigi, Japan

**Keywords:** Lipofection, CRISPR, Cas9, In vitro fertilized zygote, Pig

## Abstract

**Objective:**

Lipofection-mediated introduction of the CRISPR/Cas9 system in porcine zygotes provides a simple method for gene editing, without requiring micromanipulation. However, the gene editing efficiency is inadequate. The aim of this study was to improve the lipofection-mediated gene editing efficiency by optimizing the timing and duration of lipofection.

**Results:**

Zona pellucida (ZP)-free zygotes collected at 5, 10, and 15 h from the start of in vitro fertilization (IVF) were incubated with lipofection reagent, guide RNA (gRNA) targeting *GGTA1*, and Cas9 for 5 h. Lipofection of zygotes collected at 10 and 15 h from the start of IVF yielded mutant blastocysts. Next, ZP-free zygotes collected at 10 h from the start of IVF were incubated with lipofection reagent, gRNA, and Cas9 for 2.5, 5, 10, or 20 h. The blastocyst formation rate of zygotes treated for 20 h was significantly lower (*p* < 0.05) than those of the other groups, and no mutant blastocysts were obtained. Moreover, the mutation rates of the resulting blastocysts decreased as the incubation time increased. In conclusion, a lipofection-mediated gene editing system using the CRISPR/Cas9 system in ZP-zygotes is feasible; however, further improvements in the gene editing efficiency are required.

**Supplementary Information:**

The online version contains supplementary material available at 10.1186/s13104-021-05800-8.

## Introduction

Pigs are excellent laboratory models for medical research owing to their similarity to humans, both anatomically and physiologically. Various techniques, such as somatic cell nuclear transfer, and microinjection-mediated editing using the clustered regularly interspaced short palindromic repeats (CRISPR)/CRISPR-associated protein 9 (Cas9) system, are widely used to generate gene-modified porcine embryos [1]. However, these strategies require sophisticated techniques and/or specialized equipment for micromanipulation, in addition, the results depend on the operator’s skill. Electroporation-mediated introduction of CRISPR/Cas9 system enables efficient gene editing in porcine zygotes without sophisticated techniques [2], however, this strategy also requires specialized equipment. Therefore, simplified and equipment-free methods are needed for the large-scale and general production of genetically modified pigs.

Lipofection, defined as liposome-mediated transfection, involves the introduction of foreign nucleic acids into mammalian cells using lipophilic reagents that increase the cellular uptake of polynucleotides [3, 4]. Liposomes containing nucleic acids enter cells by endocytosis [5]. Lipofectamine 2000 is a cationic liposome-based reagent with sufficient capacity for transfection in vitro [6]. We have previously demonstrated that a lipofection-mediated gene editing system using Lipofectamine 2000 and CRISPR/Cas9 components can be applied to zona pellucida (ZP)-free oocytes and embryos in pigs [7]. However, the optimal conditions for lipofection-mediated nucleic acid transfection depend on the cell line [3, 8], and an efficient protocol has not been established for pig zygotes. The adequate timing of the introduction of the CRISPR/Cas9 system into zygotes should be optimized to improve gene editing efficiency. Moreover, the incubation duration of lipofection reagent affects the efficiency of transfection [3]. However, whether the duration has an impact on the lipofection-mediated system is also unclear in zygotes. The factors affecting the efficiency of lipofection-mediated gene editing for porcine zygotes should be further examined.

In this study, two parameters affecting the efficiency of lipofection-mediated gene editing were evaluated: the timing of the introduction of the CRISPR/Cas9 system into zygotes and the duration of incubation with lipofection reagent using porcine ZP-free zygotes.

## Main text

### Materials and methods

#### Oocyte collection, in vitro maturation(IVM), in vitro fertilization (IVF), and in vitro culture (IVC)

Oocyte collection, IVM, IVF, and IVC were performed as described previously [9]. Briefly, pig ovaries were obtained from prepubertal crossed gilts at a local slaughterhouse. Cumulus-oocyte complexes (COCs) were collected and cultured in maturation medium. After IVF, the putative zygotes were cultured in porcine zygote medium (PZM-5; Research Institute for the Functional Peptides Co.) until lipofection-mediated gene editing. After lipofection, zygotes were cultured for 3 days in PZM-5. Subsequently, the embryos were cultured in porcine blastocyst medium (PBM; Research Institute for the Functional Peptides Co.) for 4 days.

#### Lipofection-mediated introduction of the CRISPR/Cas9 system

ZP-free zygotes were prepared before lipofection-mediated gene editing. The zygotes were exposed to 0.5% (w/v) actinase-E (Kaken-Seiyaku Corp., Tokyo, Japan) in Dulbecco’s PBS (Nissui Pharmaceutical, Tokyo, Japan) for 20–30 s, transferred to PZM-5 without actinase-E, freed completely from their ZP by gentle pipetting, and then subjected to lipofection-mediated gene editing.

Lipofection solution was prepared by diluting 2 µL of Lipofectamine 2000 (Thermo Fisher Scientific) with 8 µL of Nuclease-Free Duplex Buffer (IDT, Integrated DNA Technologies, Coralville, IA, USA) and then mixing with 10 µL of Nuclease-Free Duplex Buffer containing 200 ng/µL gRNA (Alt-R CRISPR crRNAs and tracrRNA, chemically modified and length-optimized variants of native guide RNAs purchased from IDT) targeting *GGTA1* and 600 ng/µL Cas9 protein (Guide-it Recombinant Cas9, Takara Bio, Shiga, Japan). Then, 20 µL of lipofection solution was added to 180 µL of PZM-5 and used for lipofection-mediated gene editing in ZP-free zygotes. As a control, ZP-intact zygotes were cultured for 7 days without gene editing.

#### Analysis of the targeted gene sequence in embryos

Genomic DNA was isolated from blastocysts by boiling in 50 mM NaOH solution. After neutralization, the genomic regions flanking the gRNA target sequences were amplified by polymerase chain reaction (PCR) with the following specific primers for *GGTA1*: 5′-AGTCAGGATGCTTCCCCTTT-3′ (forward) and 5′-AAGCTGGTGACTTGGCTGAT-3′ (reverse). The PCR products were extracted by agarose gel electrophoresis using a Fast Gene Gel/PCR Extraction Kit (Nippon Genetics, Tokyo, Japan). The targeted genomic regions of the PCR products were directly sequenced by Sanger sequencing using the BigDye Terminator Cycle Sequencing Kit version 3.1 (Thermo Fisher Scientific) and an ABI 3500 genetic analyzer (Applied Biosystems, Foster City, CA, USA). The TIDE (tracking of indels by decomposition) bioinformatics package was used to determine the blastocyst genotype [10]. Blastocysts that carried no wild-type (WT) alleles were classified as biallelic mutants, those that carried a mutation and the WT sequence were classified as mosaics, and those that carried only the WT sequence were classified as WT. The editing rate was defined as the ratio of the number of gene-edited blastocysts to the total number of sequenced blastocysts. The editing efficiency was defined as the proportion of indel events in blastocysts carrying the mosaic or biallelic mutation products.

#### Experimental design

The gRNA targeting *GGTA1*, used for the production of *GGTA1*-deficient pigs [11], was used to investigate the gene editing of in vitro-fertilized porcine zygotes by lipofection in the following experiments.

#### Experiment 1

Zygotes were collected at 5, 10, and 15 h from the start of IVF. After ZP removal, 20 µL of lipofection solution was added to 180 µL of PZM-5 containing approximately 50 ZP-free zygotes and then the zygotes were placed in an incubator for 5 h. After co-incubation, zygotes were cultured for 7 days to assess the developmental competence and genotype of blastocysts, as described above. As a control, the ZP-intact zygotes were cultured for 7 days without lipofection treatment.

#### Experiment 2

Zygotes were collected at optimal time points with respect to the gene-editing efficiency determined in Experiment 1. Then, 20 µL of lipofection solution was added to 180 µL of PZM-5 containing approximately 50 ZP-free zygotes and they were co-incubated for 2.5, 5, 10, or 20 h, then cultured for 7 days. The ZP-free zygotes cultured for 7 days without lipofection were classified as the 0 h group (Control).

#### Statistical analysis

The percentages of embryos that developed to the blastocyst stage and mutation efficiency were subjected to arcsine transformation before analyses. Statistical significance was inferred from analysis of variance (ANOVA) followed by Fisher's protected least significant difference (PLSD) tests using STATVIEW (Abacus Concepts, Inc., Berkeley, CA, USA). The percentages of mutated blastocysts were evaluated using chi-squared tests with Yates’ correction. *p* ≤ 0.05 was regarded as significant.

### Results

#### Experiment 1

**Fig. 1:**
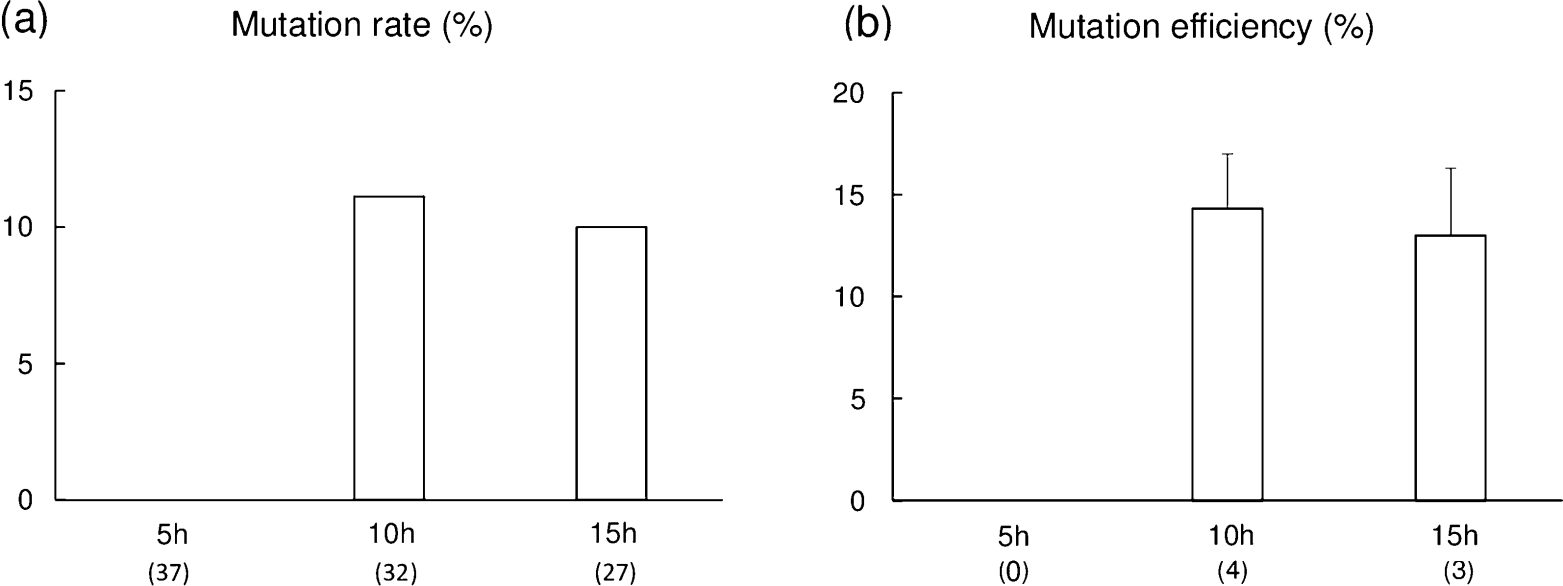
Mutation rate (**a**) and mutation efficiency (**b**) of blastocysts derived from zona pellucida (ZP)-free zygotes subjected to lipofection mediated gene editing with gRNA targeting *GGTA1*. Zygotes were collected at 5 h, 10 h, or 15 h from the start of in vitro fertilization and incubated with Lipofectamine 2000, gRNA, and Cas9 protein for 5 h. **a** Mutation rate was defined as the ratio of the number of edited blastocysts to the total number of sequenced blastocysts. **b** Mutation efficiency was estimated as the proportion of the indel mutation events in gene-edited blastocysts. Numbers within parentheses indicate the total number of blastocysts examined. Each bar represents the mean ± SEM

The blastocyst formation rates did not differ significantly with respect to the timing of lipofection (Additional file 1: Table S1). Lipofection in zygotes collected at 10 and 15 h from the start of IVF resulted in the introduction of mosaic mutations in blastocysts. However, the mutation rates (Fig. 1a) and mutation efficiency (Fig. 1b) did not differ statistically between the two time points (11.1% vs. 10.0% and 14.3% vs. 13.0%, respectively). There were no mutant blastocysts derived from treated zygotes collected at 5 h from the start of IVF.`

#### Experiment 2

**Fig. 2:**
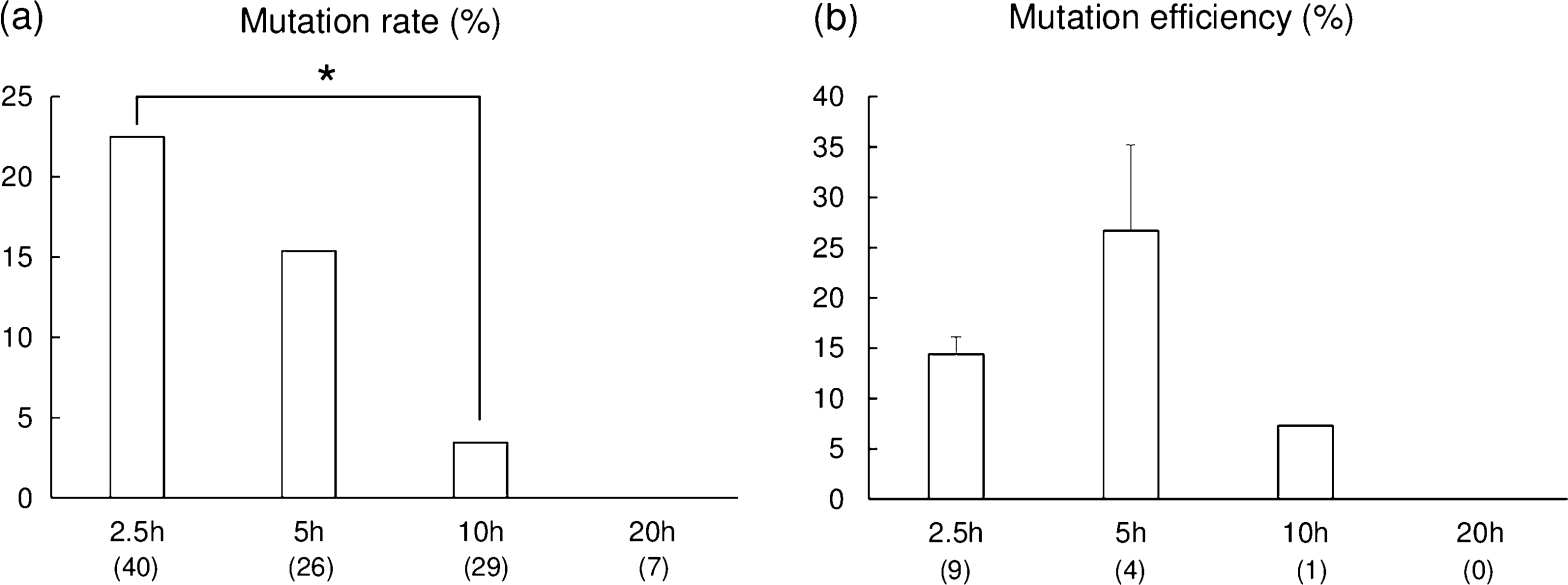
Mutation rate (**a**) and mutation efficiency (**b**) of blastocysts derived from zona pellucida (ZP)-free zygotes subjected to lipofection-mediated gene editing with gRNA targeting *GGTA1*. Zygotes were collected at 10 h from the start of in vitro fertilization and incubated with Lipofectamine 2000, gRNA, and Cas9 for 2.5 h, 5 h, 10 h, or 20 h. The mutation rate was defined as the ratio of the number of edited blastocysts to the total number of sequenced blastocysts. Mutation efficiency was estimated as the proportion of the indel mutation events in gene-edited blastocysts. Numbers within parentheses indicate the total number of blastocysts examined. Each bar represents the mean ± SEM. *p < 0.05

Zygotes collected at 10 h from the start of IVF were incubated with lipofection reagent, gRNA, and Cas9 for various durations. The blastocyst formation rate for zygotes treated for 20 h was significantly lower (*p* < 0.05) than those of the other groups, and no mutant blastocysts were obtained (Table 1). The proportion of blastocysts carrying mutations decreased as the incubation time increased (22.5%, 15.4%, and 3.4% in the 2.5, 5, and 10 h incubation groups) (Fig. 2a) and was significantly higher for the 2.5 h group (*p* < 0.05) than for the 10 h group. The mutation efficiency in resulting blastocysts did not differ significantly (14.4%, 26.7%, and 7.3% in the 2.5, 5, and 10 h incubation groups) (Fig. 2b). All mutant blastocysts carried mosaic genotypes.

**Table 1:** Development of zona pellucida (ZP)-free zygotes subjected to lipofection-mediated gene editing with gRNA targeting *GGTA1* for various incubation durations

Incubation duration*	No. of zygotes examined	No. (%) of zygotes developed to blastocysts
0 h (Control)	226	34 (15.1 ± 1.9)^a^
2.5 h	218	34 (15.5 ± 2.2)^a^
5 h	217	26 (11.7 ± 2.2)^a^
10 h	215	28 (12.8 ± 3.9)^a^
20 h	223	7 ( 3.0 ± 1.9)^b^

All experiments were repeated five times. Percentage data are expressed as means ± SEM

*ZP-free zygotes were collected at 10 h from the start of IVF and exposed to Lipofectamine 2000 for 0 h (Control), 2.5 h, 5 h, 10 h, or 20 h and then cultured for 7 days

^a,b^ Values with different superscript letters in the same column are significantly different (*p* < 0.05)

### Discussion

Techniques to generate genetically modified porcine embryos, e.g., microinjection, electroporation, and somatic cell nuclear transfer, generally require specialized equipment. We have previously demonstrated that lipofection-mediated gene editing by the CRISPR/Cas9 system, without specialized equipment, can be performed in ZP-free oocytes and embryos [7]. In the present study, to improve the gene-editing efficiency, we evaluated two parameters: the timing of the introduction of the CRISPR/Cas9 system into ZP-free zygotes and the duration of lipofection treatment.

We optimized the timing of the introduction of the CRISPR/Cas9 system into zygotes by lipofection. There were no significant differences in blastocyst formation rates when zygotes were incubated with or without lipofection reagent. We previously found that lipofection does not affect the developmental competence of porcine oocytes during IVF but affects that of embryos collected at 24–29 h from the start of IVF [7], suggesting that developmental competence after lipofection is affected by the embryonic stage. Although the blastocysts derived from zygotes treated with Lipofectamine 2000 at 10 and 15 h from the start of IVF had mutations, blastocysts derived from zygotes treated at 5 h lacked mutations. In mouse zygotes, chromatin is loosened during the pronucleus stage [12, 13], presumably increasing the accessibility of Cas9, resulting in successful gene editing. Therefore, lipofection treatment during pronucleus formation may be effective for the generation of gene-edited porcine embryos. In porcine in vitro fertilized oocytes, few zygotes with a pronucleus with decondensed chromosomes and envelope are observed at 6 h after insemination [14], supporting our results showing a low editing efficiency at 5 h after insemination.

Extensive research has focused on lipofection reagents as a vector for introducing nucleic acids in vitro; however, various parameters affect the efficiency of liposome-mediated transfection [4, 6]. The duration of incubation with transfection reagents affects gene delivery [3]; however, Lipofectamine 2000 has cytotoxicity [15]. Lipofectamine 2000 is a 3:1 (w/w) liposome formulation of the polycationic lipid 2,3-dioleyloxy-*N*-[2(sperminecarboxamide)ethyl]-*N*,*N*-dimethyl-1-propanaminium trifluoroacetate (DOSPA) and dioleoyl phosphatidylethanolamine (DOPE). DOPE is a neutral lipid with a high transfection efficiency and low toxicity [16]. Multivalent cationic compounds, such as DOSPA, accumulate in various intracellular organelles after the dissociation of nucleic acids, resulting in cell death, cell shrinking, vacuolization of the cytoplasm, and reduced mitoses [16–18].

Additionally, we found that blastocyst mutation rates decreased as the duration of incubation with Lipofectamine 2000 increased. The rate of blastocysts carrying mutations derived from the 10 h incubation group was significantly lower than that of the 2.5 h incubation group, and no mutant blastocysts were obtained in the 20 h incubation group. As the duration of the co-incubation of Lipofection reagent and ZP-free zygotes increase, developmental competence and gene editing efficiency decreased, which could be explained by the toxic effects of cationic compounds. Therefore, the long-term introduction of a large amount of biochemically stable cationic amphiphiles into the cell membrane may have adverse effects on cell function, thereby restricting nucleic acid transfer and embryonic development.

Porcine in vitro production systems including IVM, IVF, and IVC, have been established as stable methods and allow the preparation of large numbers of embryos derived from the ovaries of slaughtered pigs. However, gene modification in porcine embryos using previous methods is generally limited by the skills of operators and the research environment, including equipment in laboratories. Lipofection-mediated gene editing systems have potential for the simple, rapid, and stable large-scale production of gene-modified pigs as a micromanipulation-free strategy independent of skills and experience. Gene-modified pigs are promising experimental animal models for human diseases and as donors for pig-to-human xenotransplantation [19, 20]. The lipofection-mediated gene editing system described here still has some limitations including the necessity of ZP removal and insufficient gene-editing efficiency, which has higher risks of mosaicism when compared to microinjection- and electroporation-meditated gene-editing [2, 21, 22]. Nevertheless, continuous optimization of this gene editing system will substantially improve the value of pigs as experimental animals, particularly in unequipped laboratories.

In conclusion, we verified the feasibility of a lipofection-mediated gene editing system without microinjection or electroporation for inserting gRNA and Cas9 into ZP-free zygotes, particularly when zygotes collected at 10 h from the start of IVF were treated with Lipofectamine 2000 for 2.5 h to generate gene-edited blastocysts with high efficiency.

## Limitations

In our lipofection-mediated gene editing system for zygotes, the gene editing efficiency was still insufficient compared with those of microinjection and electroporation-meditated gene-editing [2, 22]. Microinjection- and electroporation-mediated gene editing efficiencies are affected by the concentrations of CRISPR/Cas9 components and the embryonic stage [21, 23]. Additionally, the DNA: lipofection reagent ratio affects the transfection efficiency [6], indicating that the ratio of CRISPR/Cas9 components to lipofection reagent can influence the gene editing efficiency. It is necessary to further optimize the transfection protocol for the generation of mutant embryos using a lipofection-mediated gene editing system.

## Supplementary Information

**Additional file 1: Table S1.** Development of zona pellucida (ZP)-free zygotes subjected to lipofection-mediated gene editing with gRNA targeting GGTA1 at different time points from the start of IVF.

## Data Availability

The datasets used and/or analyzed during the current study are available from the corresponding author on reasonable request.

## Abbreviations

ANOVA: Analysis of variance
CRISPR: The clustered regularly interspaced short palindromic repeats
Cas: CRISPR-associated gene system
COCs: Cumulus-oocyte complexes
IVF: In vitro fertilization
IVM: in vitro maturation
PBM: Porcine blastocyst medium
PCR: Polymerase chain reaction
PFM: Porcine fertilization medium
PZM-5: Porcine zygote medium
TIDE: Tracking of indels by decomposition
WT: Wild-type
ZP: Zona pellucida
