# Supplementary material for: Timing and duration of lipofection-mediated CRISPR/Cas9 delivery into porcine zygotes affect gene-editing events
Source: BMC Res Notes. 2021 Oct 9;14:389. doi: 10.1186/s13104-021-05800-8 (PMC8502333; doi:10.1186/s13104-021-05800-8)
Supplement: Supplementary file 1 — Additional file 1: Table S1. Development of zona pellucida (ZP)-free zygotes subjected to lipofection-mediated gene editing with gRNA targeting GGTA1 at different time points from the start of IVF. [file 13104_2021_5800_MOESM1_ESM.docx]

Supplementary Table 1. Development of zona pellucida (ZP)-free zygotes subjected to lipofection-mediated gene editing with gRNA targeting *GGTA1* at different time points from the start of IVF*

| Timing** | No. of zygotes examined | No. (%) of zygotes developed to blastocysts |
| --- | --- | --- |
| Control | 310 | 46 (14.2 ± 1.5) |
| 5 h | 316 | 38 (12.1 ± 1.8) |
| 10 h | 305 | 37 (12.0 ± 1.4) |
| 15 h | 316 | 32 (10.2 ± 2.3) |

* All experiments were repeated seven times. Percentage data are expressed as means ± SEM.

** Zygotes were collected at 5 h, 10 h, or 15 h from the start of IVF, followed by ZP removal, exposure to Lipofectamine 2000 for 5 h, and culture for 7 days. As a Control, ZP-intact zygotes were cultured for 7 days without lipofection treatment.
